# P-275. Geographic Variations of HIV Specialist Shortages: An Observational Study to Support the 90-90-90 HIV Treatment Goals in the US

**DOI:** 10.1093/ofid/ofaf695.496

**Published:** 2026-01-11

**Authors:** Dona Khoshabafard, Juan Yang, Julia Green, Gina Brown, Amy Weinberg, Li Tao

**Affiliations:** Gilead Sciences, Inc., Foster City, California; Gilead Sciences, Inc. Foster City, CA, USA, Foster City, California; Gilead Sciences, Inc., Foster City, California; Gilead Sciences, Inc., Foster City, California; Gilead Sciences, Inc., Foster City, California; Gilead Sciences, Inc. Foster City, CA, USA, Foster City, California

## Abstract

**Background:**

Access to HIV care is essential, however significant variability and shortages in experienced-HIV-care providers persist in the US.

**Figure:**
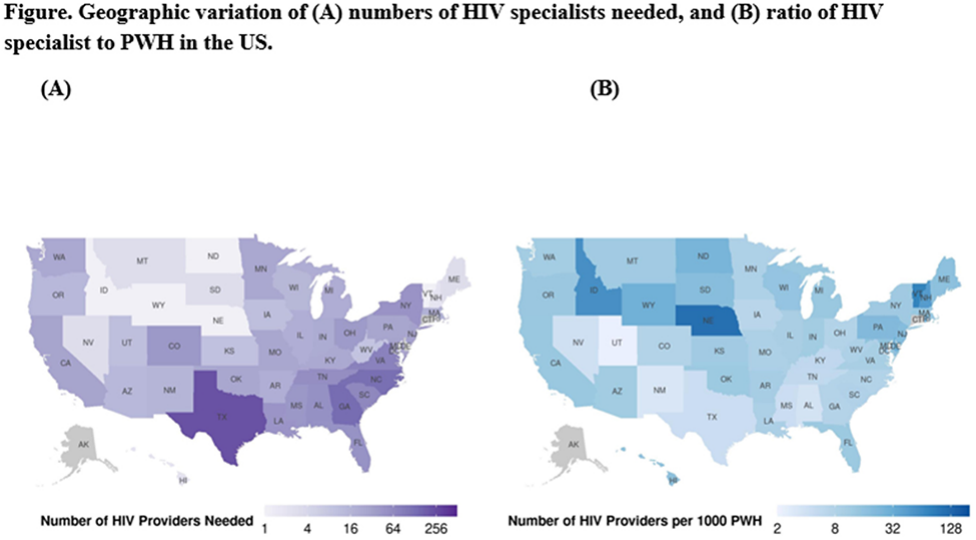

**Methods:**

Experienced-HIV-care providers delivering care to ≥25 people with HIV (PWH) between 2022 and 2024 were identified using the IQVIA LAAD database. HIV specialists were linked to multiple county-level databases: demographic data from the 2020 Census, health resource data from the Health Resources and Services Administration, and HIV surveillance and social determinant data from AIDSVu. Counties with an HIV specialist shortage were defined as those with a specialist-to-PWH ratio (SPR) lower than the average SPR among counties achieving a 90% HIV treatment goal in linkage-to-care, receipt of care, or viral suppression. For counties not meeting the HIV-care-goal, the number of specialists needed to achieve the 90% targets was calculated by multiplying the number of PWH by the difference between the targeted and observed SPRs.

**Results:**

A total of 21,003 HIV specialists were identified in 2,972 counties. Most practice in family medicine (29%), infectious disease (23%), or internal medicine (19%). The majority practiced in urban areas (98%), predominantly in mixed race/ethnicity (51%) and non-Hispanic White neighborhoods (34%), 4% practiced in non-Hispanic Black and 8% in Hispanic neighborhoods. 13% of providers practiced in low-income neighborhoods, and 14% in neighborhoods with reduces insurance coverage.

The average HIV SPR was 11:1000 nationwide and 13:1000 in counties achieving the 90% target in at least one of the three HIV care quality metrics. Significant geographic variations in SPR were evident, particularly in Southern States where HIV prevalence is high and SPR was notably low (8:1000). An additional 1,565 HIV specialists are needed to meet the 90-90-90 goals in the US. This shortage unevenly distributed, notably in Texas and Georgia, requiring 236 and 137 additional HIV specialists, respectively (Figure).

**Conclusion:**

This analysis highlights socioeconomic and geographic variations in HIV provider availability, with significant shortages in some states. Strategic interventions are needed to ensure access to HIV care and address workforce shortages in regions of greatest need.

**Disclosures:**

Dona Khoshabafard, PharmD, Gilead Sciences, Inc.: Employee|Gilead Sciences, Inc.: Ownership Interest|Gilead Sciences, Inc.: Stocks/Bonds (Public Company) Juan Yang, PhD, Gilead Sciences, Inc.: Employee and shareholder Julia Green, MS, APRN, AGNP-C, AAHIVE, Gilead Sciences, Inc.: Employee|Gilead Sciences, Inc.: Stocks/Bonds (Public Company) Gina Brown, MD, Gilead Sciences, Inc.: Employee|Gilead Sciences, Inc.: Stocks/Bonds (Public Company) Amy Weinberg, DNP, MS, Gilead Sciences, Inc.: Employee|Gilead Sciences, Inc.: Stocks/Bonds (Public Company) Li Tao, PhD, Gilead Sciences, Inc.: Employee and shareholder

